# Supplementary material for: Slc7a5 regulates Kv1.2 channels and modifies functional outcomes of epilepsy-linked channel mutations
Source: Nat Commun. 2018 Oct 24;9:4417. doi: 10.1038/s41467-018-06859-x (PMC6200743; doi:10.1038/s41467-018-06859-x)
Supplement: Supplementary file 1 — Supplementary Information [file 41467_2018_6859_MOESM1_ESM.pdf]

Supplementary Figures For:

Slc7a5 regulates Kv1.2 channels and modifies functional outcomes of epilepsy-linked channel mutations

Victoria A. Baronas, Runying Y. Yang, Luis Carlos Morales, Simonetta Sipione, Harley T. Kurata

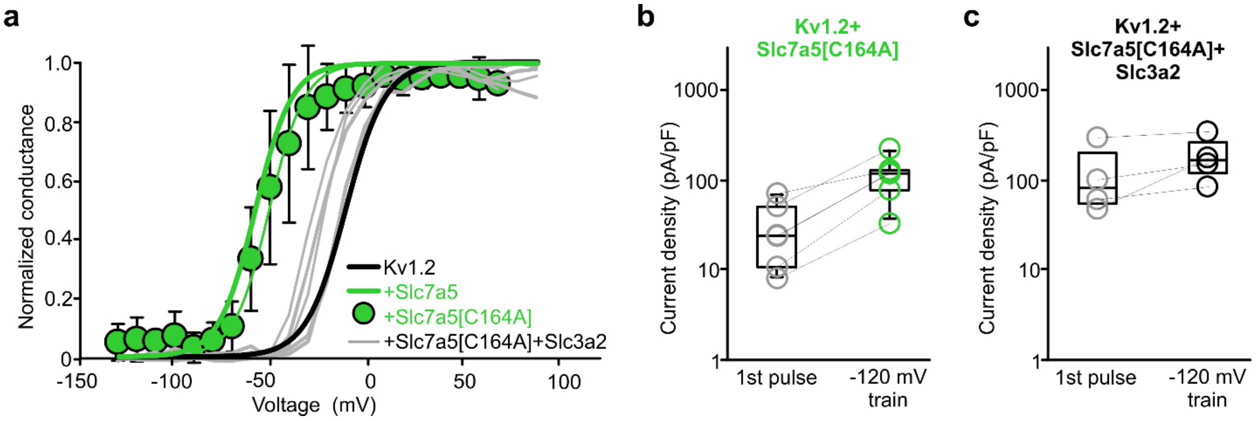

Supplementary Figure 1. Slc7a5[C164A] preserves gating shift and current suppression of Kv1.2. Kv1.2, Slc7a5[C164A], Slc3a2 (2:1:2 transfections ratios) were expressed in mouse *Itk*-fibroblast cells in the indicated combinations. (a) Mean conductance-voltage relationship for Slc7a5[C164A] + Kv1.2 ($V_{1/2}$ = 56 ± 8 mV, k = 10 ± 2 mV) and individual conductance-voltage relationships for 5 cells transfected with Kv1.2, Slc7a5[C164A] and Slc3a2 (grey lines (mean $V_{1/2}$ = -18 ± 7 mV, k = 11 ± 3 mV). (b,c) Disinhibition after a train with a -120 mV holding voltage (identical to Fig. 3) is shown on a cell by cell basis (7-fold for Kv1.2 + Slc7a5[C164A], 1.5-fold for Kv1.2 + Slc7a5[C164A]+Slc3a2). The extent of rescue by co-expression with Slc3a2 varies on a cell-to-cell basis.

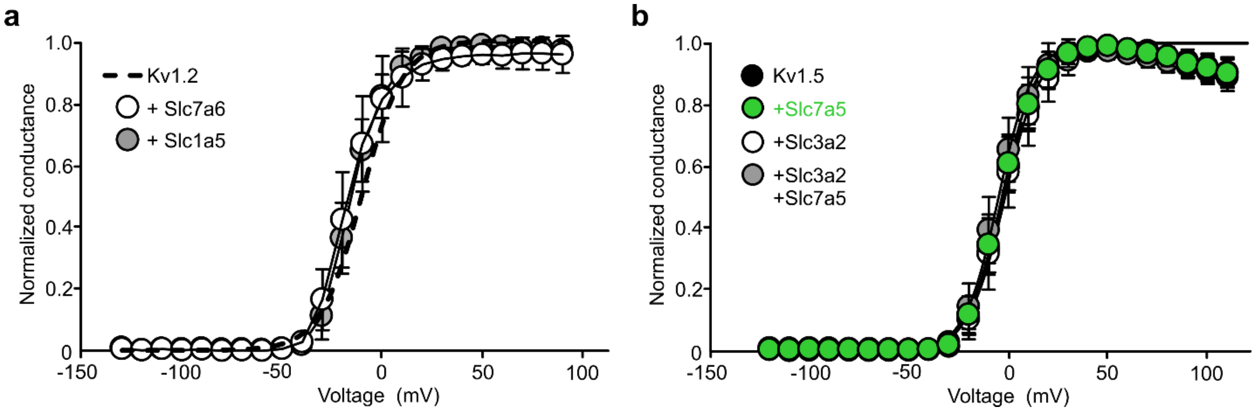

Supplementary Figure 2. Other Slc7 and Kv1 subtypes do not alter gating. (a) Conductance-voltage relationships for Kv1.2 channels co-expressed with Slc7a6 (1:1) or Slc1a5 (1:1) (+Slc7a6 $V_{1/2}$ = -15 ± 7 mV, k = 9.7 ± 2.3 mV; +Slc1a5 $V_{1/2}$ = -14 ± 5 mV, k = 9.3 ± 2.0 mV). (b) Kv1.5 channels were expressed alone or with Slc7a5 (1:1), Slc3a2 (1:1), or Slc7a5+Slc3a2 (1:1:1), conductance-voltage relationships were measured (Kv1.5 $V_{1/2}$ = -2.7 ± 2.6 mV, k = 9.0 ± 1.8 mV; +Slc7a5 $V_{1/2}$ = -5 ± 4.6 mV, k = 8.7 ± 1.8 mV; +Slc3a2 $V_{1/2}$ = -1.7 ± 5.0 mV, k = 9.3 ± 1.6 mV; +Slc7a5+Slc3a2 $V_{1/2}$ = -3.2 ±3.9 mV, k= 8.9 ± 1.4 mV). Data presented are mean ± s.d., n=4. No statistical differences were detected (ANOVA).

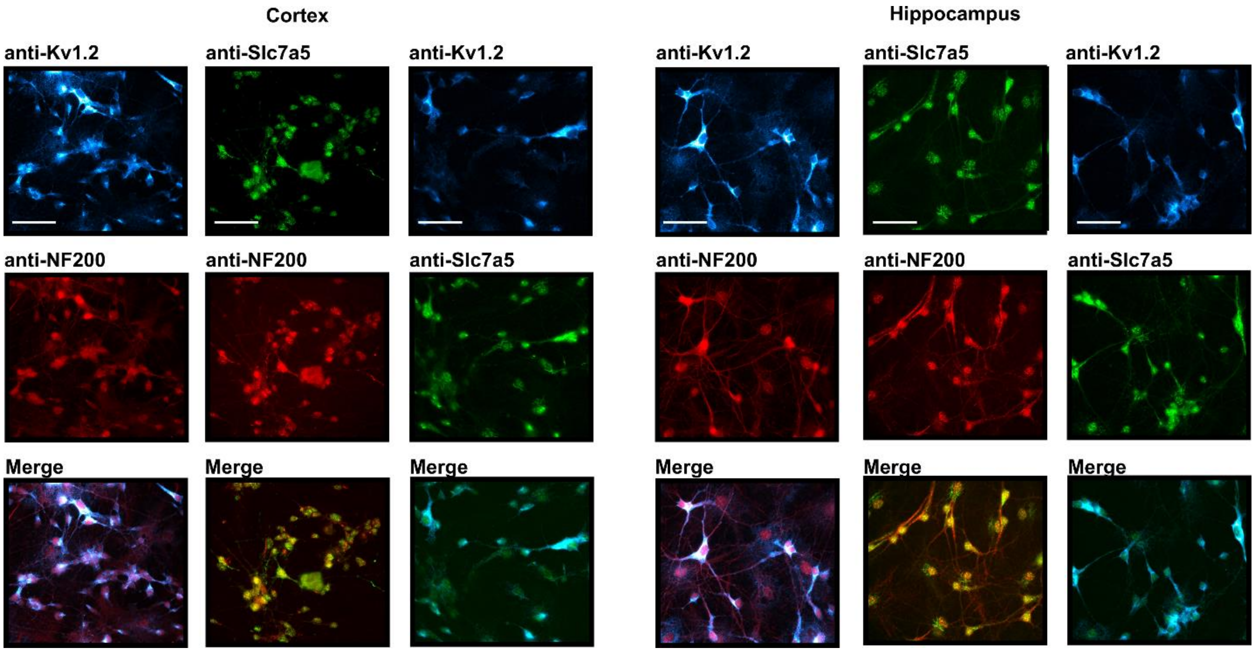

Supplementary Figure 3. Slc7a5 and Kv1.2 are expressed in dissociated rat cortical and hippocampal neurons. Hippocampal and cortical neurons were isolated from P0-2 rat pups and cultured for 7 days *in vitro*. Neurons were fixed and stained for either endogenous Slc7a5 or Kv1.2 and endogenous NF-200, as indicated to confirm neuronal phenotype. Additionally, neurons were stained for both Slc7a5 and Kv1.2 to demonstrate that both are expressed in the same cells. These images are complimentary to images in Fig. 6f, including neuronal markers to confirm expression of Slc7a5 and Kv1.2 in dissociated neurons. Scale bars are 50 µM.

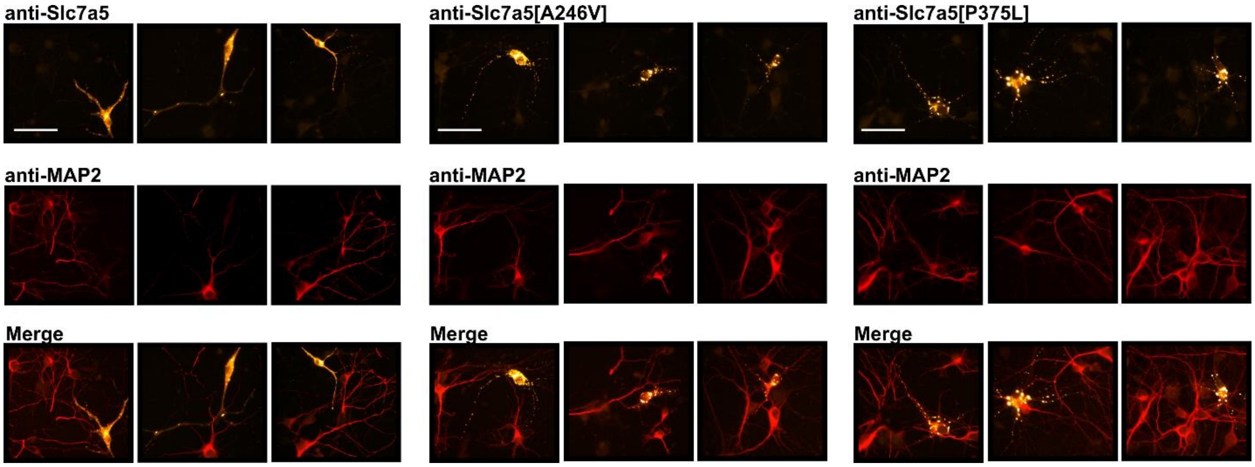

Supplementary Figure 4. Slc7a5 autism-related mutations exhibit a neuronal localization defect.

Cortical neurons were isolated from P0-2 rat pups and cultured for 7 days *in vitro*, then transfected with either N-terminally tagged mCherry-Slc7a5 WT, Slc7a5[A246V] or Slc7a5[P375L] for 3 days. At 10 days *in vitro*, neurons were fixed and co-stained for MAP2 to confirm neuronal phenotype. Multiple images were collected for each Slc7a5 mutant. Scale bars are 50 μM.

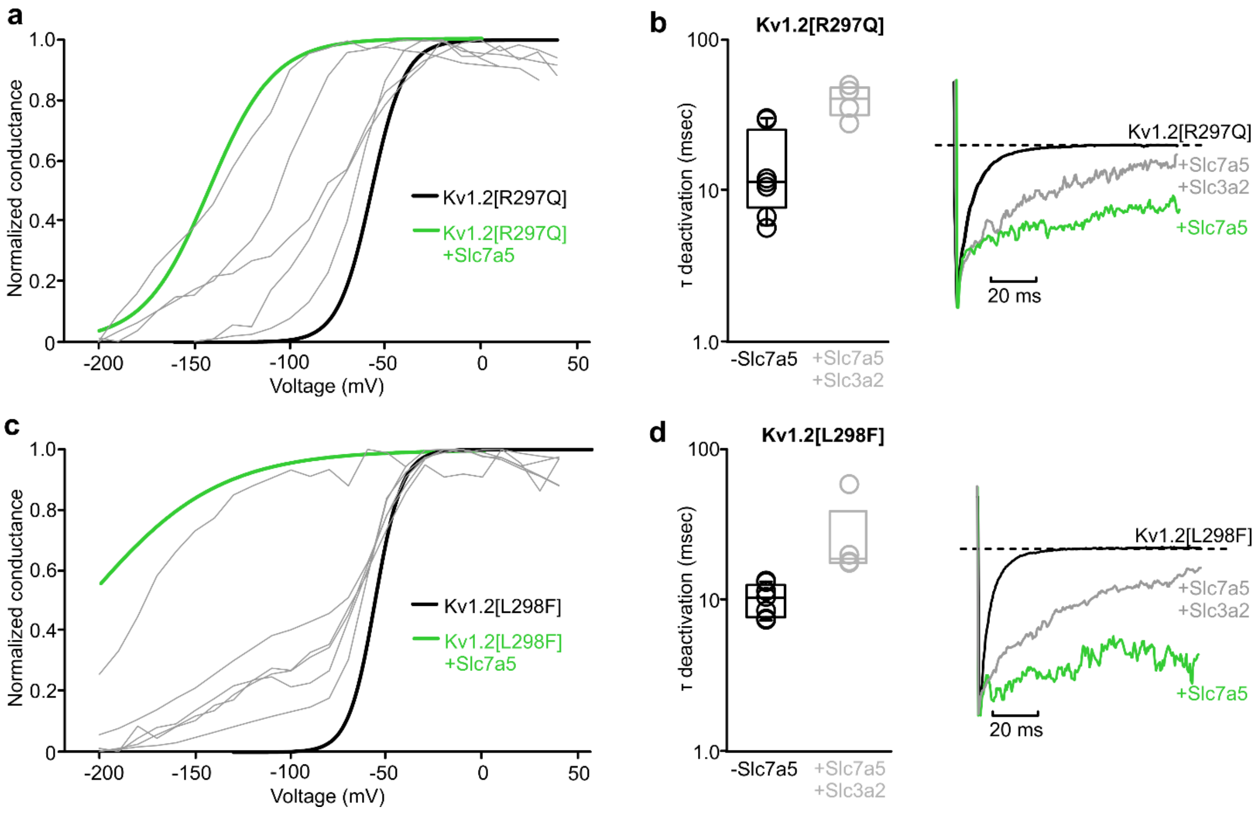

Supplementary Figure 5. Slc3a2 partially restores gating to epilepsy-linked Kv1.2 mutations. (a) Kv1.2[R297Q] or Kv1.2[L298F] channels were co-expressed with Slc7a5 + Slc3a2 (2:1:2 transfection ratio) in *Itk*- mouse fibroblast cells. Conductance-voltage relationships were gathered as described in Fig. 1 and each individual curve is shown in grey. Solid thick lines are conductance-voltage relationships for each Kv1.2 mutant (black) + Slc7a5 (green), copied from Fig. 9 for direct comparison (n=5-6). (b) Measurement of the deactivation time constant at -130 mV of Kv1.2[R297Q] was τ = 10 ± 3 ms, Kv1.2[R297Q] + Slc7a5 + Slc3a2 was τ = 28 ± 20 ms, Kv1.2[L298F] was τ = 15 ± 10 ms and Kv1.2[L298F] + Slc7a5 + Slc3a2 was τ = 40 ± 10 ms. Deactivation time constants could not be measured for the Kv1.2 mutant + Slc7a5 as channels did not close at -130 mV. (c) Representative traces of deactivation of the indicated constructs at -130 mV following a depolarization to +60 mV.

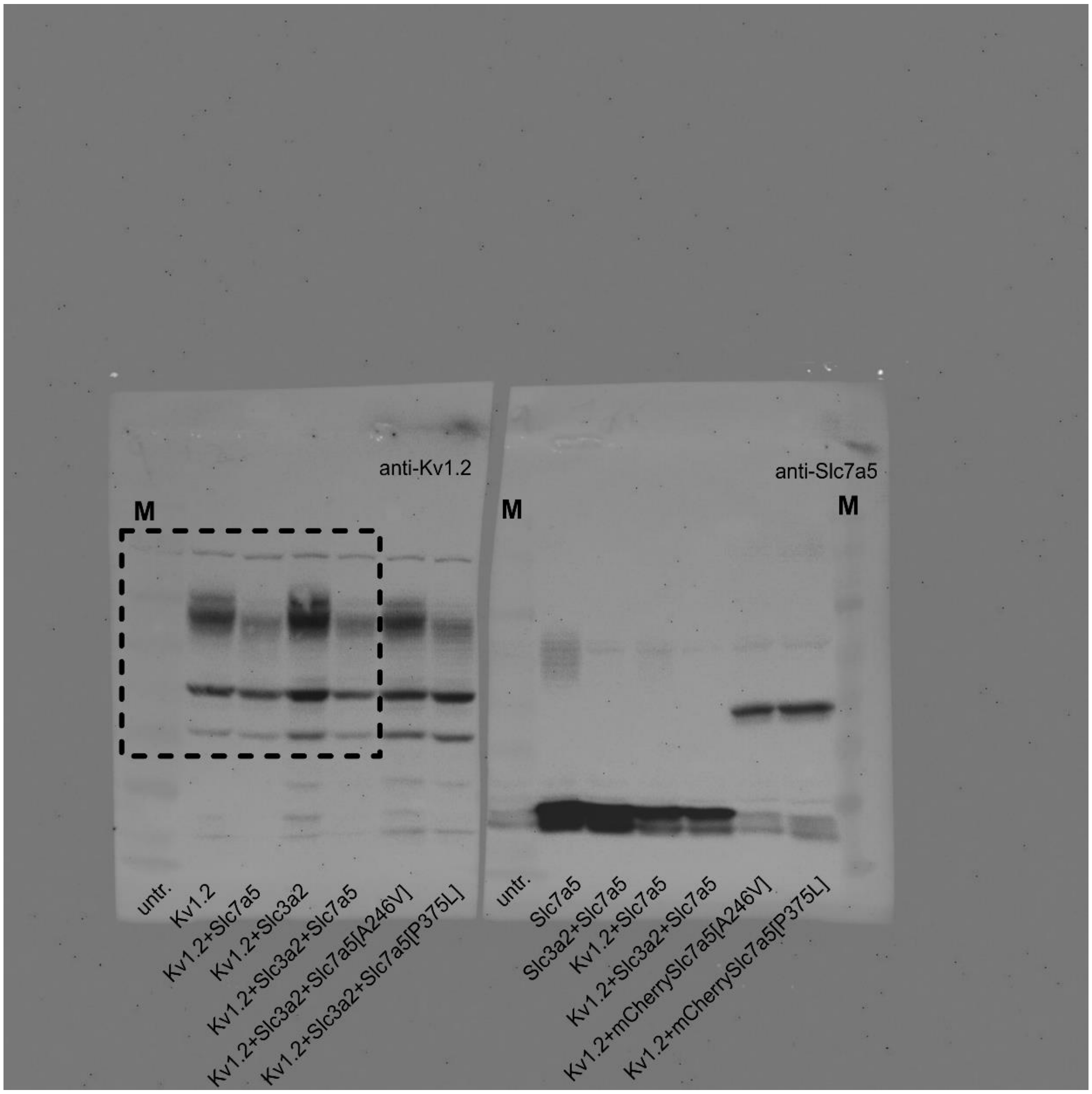

Supplementary Figure 6. Raw western blot image used for Figure 2b. Dashed lines indicate the approximate region of the blot that was used for Figure 2b.

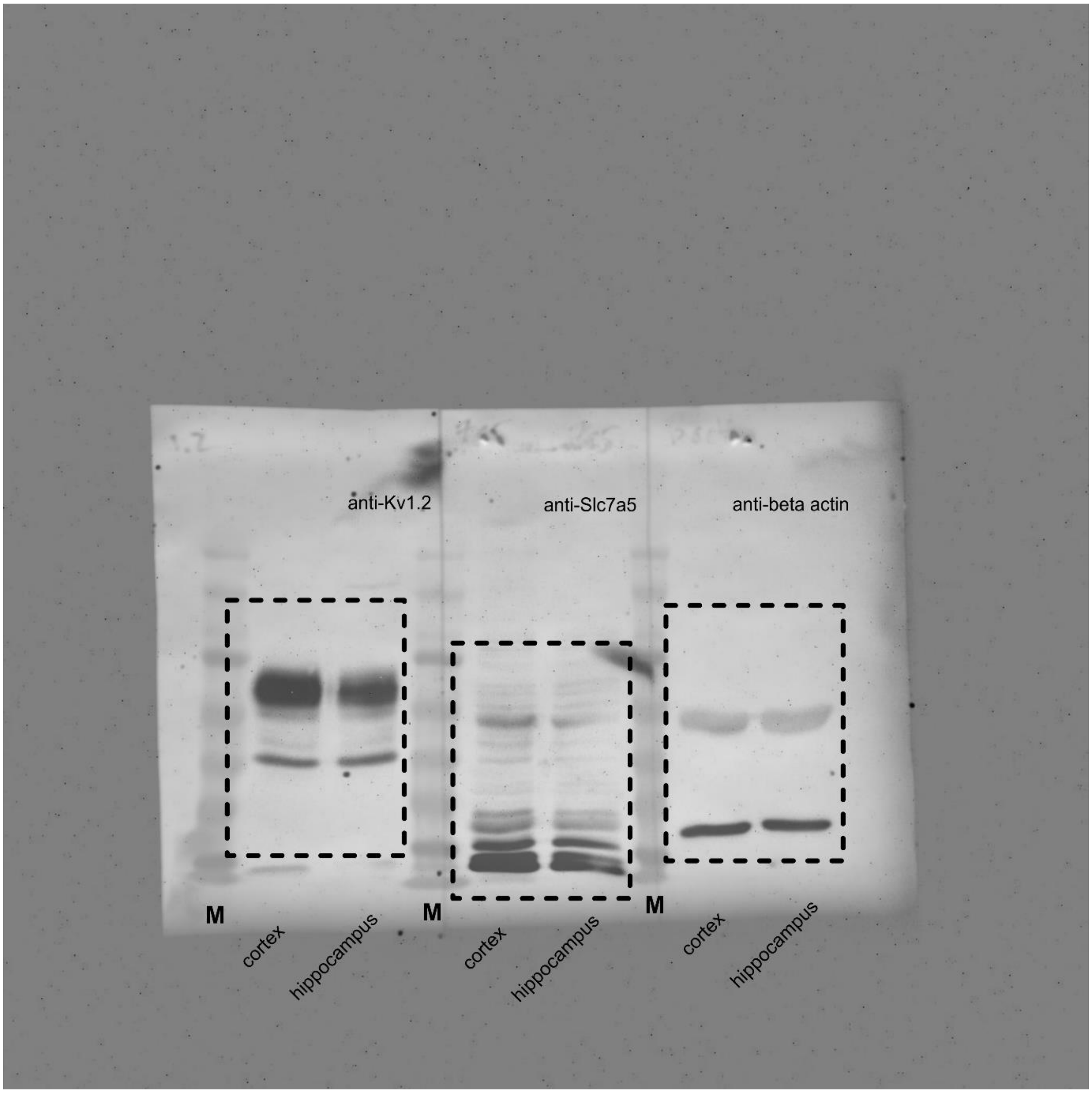

Supplementary Figure 7. Raw western blot image used for Figure 6d. Dashed lines indicate the approximate region of the blot that was used for Figure 6d.

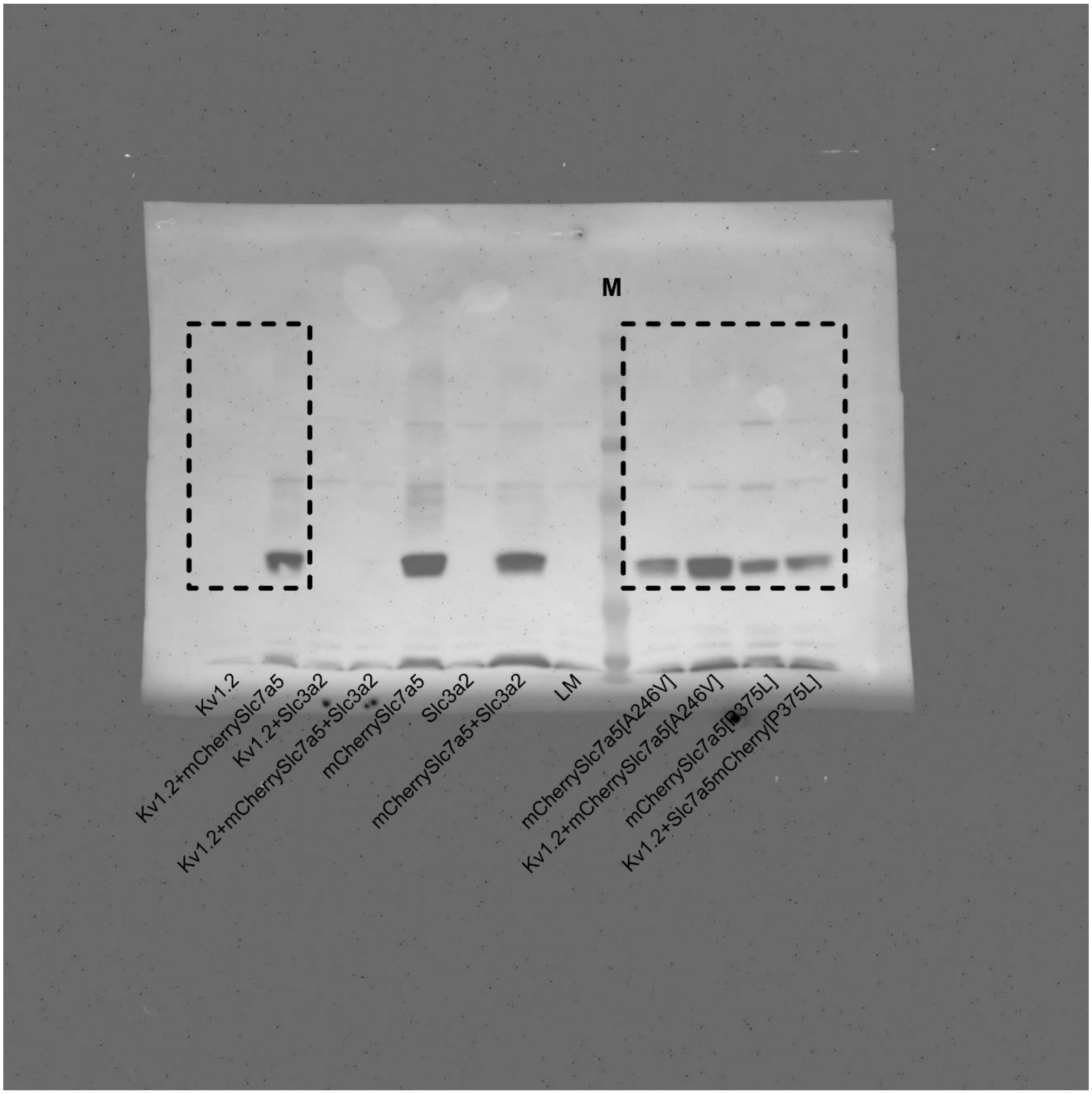

Supplementary Figure 8. Raw western blot image used for Figure 8d. Dashed lines indicate the approximate regions of the blot that was used for Figure 8d.

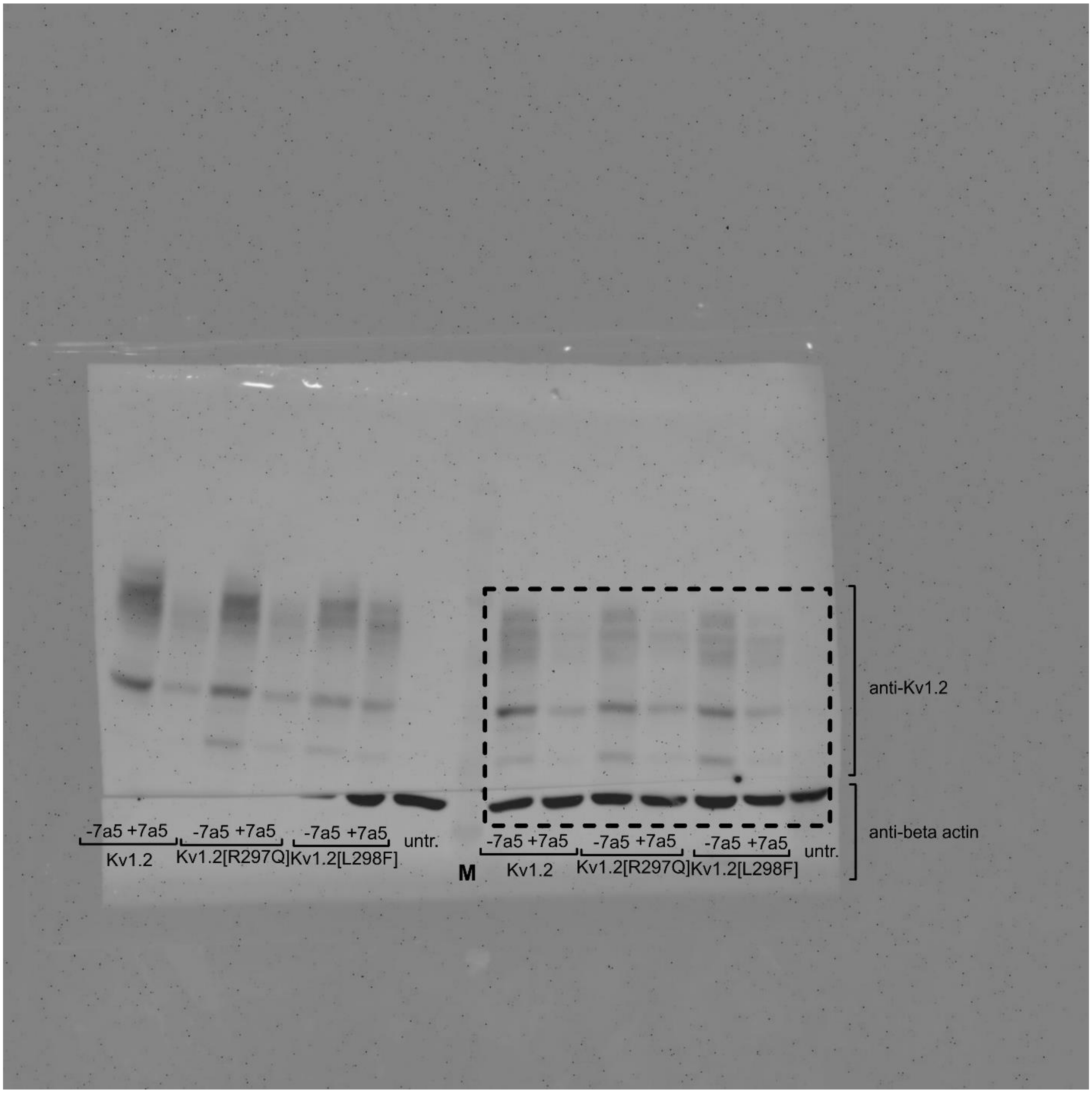

Supplementary Figure 9. Raw western blot image used for Figure 9g. Dashed lines indicate the approximate region of the blot that was used for Figure 9g.

Mutation	5' primer	3' primer
Kv1.2[V381T]	5': gga gac atg act cca act acc	3': ggt agt tgg agt cat gtc tcc
Kv1.2[I304L]	5': gtc ttt agg ctt ttc aag ttg	3': caa ctt gaa aag cct aaa gac
Kv1.2[S308T]	5': gtc ttt agg ctt ttc aag ttg	3': caa ctt gaa aag cct aaa gac
Kv1.2[R297Q]	5': cgt gtc atc cag ttg gta aga	3': tct tac caa ctg gat gac acg
Kv1.2[L298F]	5': gtc atc cgg ttc gta aga gtc	3': gac tct tac gaa ccg gat gac
Slc7a5[C164A]	5': c ttc ccc acc gcc ccg gtg ccc	3': ggg cac cgg ggc ggt ggg gaa g
Slc7a5[A246V]	5': c att gtg ctg gta tta tac agc	3': gct gta taa tac cag cac aat g
Slc7a5[P375L]	5': c acc ccc gtg ctg tcc ctc gtg	3': cac gag gga cag cac ggg ggt g

Supplementary Table 1. Primers used for mutagenesis of Kv1.2 and Slc7a5.
